# Impairment of Neuroplasticity in the Dorsolateral Prefrontal Cortex by Alcohol

**DOI:** 10.1038/s41598-017-04764-9

**Published:** 2017-07-13

**Authors:** Genane Loheswaran, Mera S. Barr, Reza Zomorrodi, Tarek K. Rajji, Daniel M. Blumberger, Bernard Le Foll, Zafiris J. Daskalakis

**Affiliations:** 1 Translational Addiction Research Laboratory, Toronto Ontario, Canada; 2 Temerty Centre for Therapeutic Brain Intervention, Toronto Ontario, Canada; 3 Biobehavioural Addictions and Concurrent Disorders Laboratory (BACDRL), Schizophrenia Program; Centre for Addiction and Mental Health, Toronto Ontario, Canada; 4 University of Toronto, Toronto Ontario, Canada

**Keywords:** Long-term potentiation, Neurophysiology

## Abstract

Previous studies have demonstrated that alcohol consumption impairs neuroplasticity in the motor cortex. However, it is unknown whether alcohol produces a similar impairment of neuroplasticity in the dorsolateral prefrontal cortex (DLPFC), a brain region that plays an important role in cognitive functioning. The aim of the current study was to evaluate the effect of alcohol intoxication on neuroplasticity in the DLPFC. Paired associative stimulation (PAS) combined with electroencephalography (EEG) was used for the induction and measurement of associative LTP-like neuroplasticity in the DLPFC. Fifteen healthy subjects were administered PAS to the DLPFC following consumption of an alcohol (1.5 g/l of body water) or placebo beverage in a within-subject cross-over design. PAS induced neuroplasticity was indexed up to 60 minutes following PAS. Additionally, the effect of alcohol on PAS-induced potentiation of theta-gamma coupling (an index associated with learning and memory) was examined prior to and following PAS. Alcohol consumption resulted in a significant impairment of mean (t = 2.456, df = 13, p = 0.029) and maximum potentiation (t = −2.945, df = 13, p = 0.011) compared to the placebo beverage in the DLPFC and globally. Alcohol also suppressed the potentiation of theta-gamma coupling by PAS. Findings from the present study provide a potential neurophysiological mechanism for impairment of cognitive functioning by alcohol.

## Introduction

Alcohol consumption produces a number of neurochemical and neurophysiological changes in the brain. Acute alcohol consumption is broadly defined as consumption of any given volume of alcohol over a short period of time. Binge drinking is defined more specifically by the National Institute on Alcohol Abuse and Alcoholism (NIAAA) as “…a pattern of drinking that brings blood alcohol concentration (BAC) levels to 17.4 mM (0.08% BAC). This typically occurs after 4 drinks for women and 5 drinks for men—in about 2 hours”. (http://www.niaaa.nih.gov/)”. Multiple lines of evidence suggest that acute consumption of alcohol (including binge drinking) results in an increase in GABA_A_ receptor mediated neurotransmission^1–4^ and a decrease in NMDA receptor mediated transmission^5–7^. These aberrancies in neurotransmission with alcohol use may adversely affect neuroplasticity, which is dependent on both GABA_A_ and NMDA receptor activities^8, 9^.

Paired associative stimulation (PAS) is a transcranial magnetic stimulation (TMS) protocol that can be used to index long-term potentiation (LTP)-like neuroplasticity *in vivo*. Previous PAS studies have demonstrated that alcohol impairs neuroplasticity indexed from the motor cortex among healthy subjects^10, 11^. Our group examined the effect of alcohol intoxication (at doses of alcohol high enough to constitute heavy drinking) in 15 subjects up to 60 minutes and 24 hours following PAS in a within-subject crossover design study. Consumption of high doses of alcohol significantly suppressed LTP-like neuroplasticity at 30 minutes and 60 minutes following PAS administration compared to placebo^11^.

Findings from previous studies suggest that alcohol consumption affects neurophysiology in the dorsolateral prefrontal cortex (DLPFC) (for review, see ref. 12). The DLPFC is a region in the brain within the mesocortico-limbic pathway that is implicated in the pathophysiology of addiction^13^. The DLPFC plays an important role in reward processing to guide behaviour and mediates cognitive functioning including working memory (for review, see ref. 14). Given that neuroplasticity is the principle mechanism underlying learning and memory important in cognitive functioning^15, 16^, indexing the effect of consuming high doses of alcohol on DLPFC neuroplasticity may reveal a potential mechanism of cognitive dysfunction observed during alcohol intoxication.

Despite the important implications of neuroplasticity impairment by alcohol in the DLPFC, no study to date has examined the effect of alcohol intoxication on neuroplasticity in this region in humans *in vivo*. Our group has demonstrated that LTP-like neuroplasticity can be indexed from the DLPFC by combining PAS with electroencephalography (EEG)^17^. EEG allows for the measurement of cortical evoked activity (CEA), which is the net brain activity to a stimulus. Potentiation of CEA by PAS is therefore indicative of an increase in brain activity, reflective of LTP-like neuroplasticity. This potentiation of CEA was associated with an increase in coupling of cortical oscillations of theta amplitude and gamma phase (theta-gamma coupling)^17^, believed to be associated with the working memory function of the DLPFC^18^. This novel technique holds the promise of revealing the effect of alcohol on neuroplasticity in the DLPFC and allows for the indexing theta-gamma coupling.

The goal of our study was to examine the effect of alcohol intoxication on neuroplasticity in the DLPFC in fifteen healthy subjects using PAS with EEG. Additionally, the effect of alcohol intoxication on PAS potentiation of theta-gamma coupling was examined. By the definition provided by the NIAAA, the acute alcohol consumption in the present study would also meets criteria for a binge drinking episode.

## Materials and Methods

### Study Design

The current study was a within-subject, randomized cross-over study design consisting of a total of two study visits following enrollment into the study. These visits consisted of one study visit where subjects received the alcohol beverage and one study visit where subjects received the placebo beverage. During both study visits, PAS was administered to the DLPFC and EEG was collected. The order of the alcohol and placebo study visits was randomized with a minimum one-month washout period between beverage testing visits.

### Study Visits

**Figure 1:**
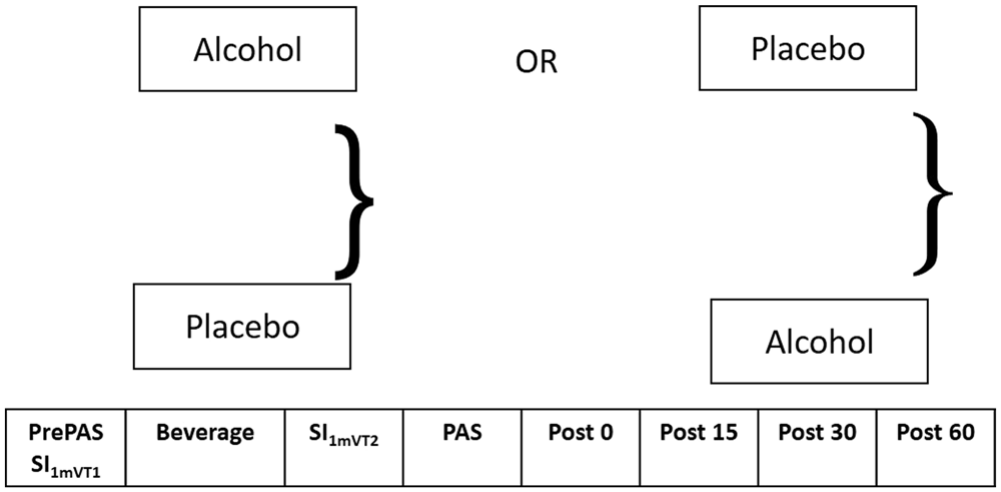
Study design. Study visits (alcohol/placebo) were randomized with a 1 month washout period between study visits. 100 TMS pulses were administered to the DLPFC at 1 mV stimulus intensity prior to beverage consumption (SI_1mVT1_). Subjects were given 15 minutes to consume the beverage. BAC was obtained every 15 minutes following beverage consumption (except during PAS). Another 100 TMS pulses were administered following beverage consumption and stimulus intensity was adjusted if necessary (SI_1mVT2_). PAS was then administered using SI_1mVT2_, followed by 100 TMS pulses also at SI_1mVT2_ at Post 0 (immediately following PAS), Post 15 (15 minutes following PAS), Post 30 (30 minutes following PAS) and Post 60 (60 minutes following PAS). EEG was collected at all time points to measure CEA.

At the beginning of all study visits, breath measures of BAC and carbon monoxide were obtained and urine drug screens were administered. Females of child bearing age were administered urine pregnancy tests before study visits. The resting motor threshold (RMT) and the stimulus intensity required to produce an average motor evoked potential (MEP) amplitude of 1 mV (1mV_T1_) was obtained. The DLPFC was identified using the F5 electrode as the marker^19^. To assess baseline cortical evoked activity (CEA), a train of 100 pulses at 0.1 Hz were delivered at stimulus intensity 1mV_T1_ pre PAS to the DLPFC (PrePAS) and EEG was collected. Subjects were then given 15 min to consume the beverage to achieve a rapid increase in BAC. Their BAC was obtained via breath measures every 15 minutes after beverage consumption and when BAC was ≥ 17.4 mM (≥0.08%), the intensity required to produce an average motor evoked potential (MEP) amplitude of 1 mV (1mV_T2_) was reassessed. To assess CEA following the beverage, another 100 pulses were administered to the DLPFC at stimulus intensity 1mV_T2_. PAS was then administered to the DLPFC. Immediately after the termination of PAS, a train of 100 pulses of TMS at 0.1 Hz frequency were delivered to the DLPFC at (Post 0), 15 minutes later (Post 15), 30 minutes post PAS (Post 30) and 60 minutes post PAS (Post 60) while EEG was collected to assess potentiation of CEA following PAS (Fig. 1).

### Subjects

Subjects were recruited through ads on-line and ads posted at the University of Toronto. All subjects provided written informed consent prior for participation in the study. All experiments were conducted in accordance with the Declaration of Helsinki and were approved by the research ethics board at the Centre for Addiction and Mental Health. Fifteen healthy drinkers participated in the study (mean age 33.42, ± 7.52, 23–46 years of age, 10 Males). Subjects had endorsed at least one heavy drinking episode (defined at 5 standard drinks for men and 4 standard drinks for women) (“NIAAA Guidelines,” 2004), within the last month, as assessed using the Alcohol Timeline Follow-Back^20^. This criteria was used to ensure that subjects would be able to tolerate the high doses of alcohol administered in the study. Subjects were between the age of 19 and 60 years of age and were of legal drinking age in Ontario, Canada. Subjects were non-smokers (had not smoked any cigarettes in the last three months) and did not meet DSM-IV criteria for any current drug abuse or dependence or any psychiatric disorders. Subjects were excluded if they had a history of seizures, neurological disease or cognitive impairment^21^ and none reported regular use of any therapeutic or recreational psychoactive drugs during the last three months. Subjects reported having had on average 4.5 ± 4.9 heavy drinking episodes. Therefore while the subjects were otherwise healthy, they would be classified as binge drinkers with an increased risk for developing AUDs. Table 1 includes the demographic information of the subjects.

**Table 1:** Subject Demographics.

	Mean ± 1 Standard Deviation (SD)
Age	32.60 ± 7.79
Sex	10 males; 5 females
Average # of years of education	16.13 ± 1.92
Average # of languages spoken	2 ± 1
Mean MMSE score	29.33 ± 0.98
Average # of heavy drinking episodes/month	4.53 ± 4.91
Average # of standard drinks/month	44.10 ± 30.27

### Beverages

Beverages were prepared as described in Loheswaran *et al*., 2015^22^. The alcohol beverage was made using 95% United States Pharmacopeia (USP) alcohol at a dose of 1.5 g/l of body water and mixed in a 1:5 ratio with orange juice and tonic water. The placebo beverage was made from an equivalent volume of orange juice and tonic water. Absolut Vodka (0.2 mL of 40% alcohol) was added to both beverages immediately prior to administering the beverage to the subject to produce the odour of alcohol but is minimal enough to not produce any additional alcohol effects. Investigators were blinded to the beverage type until after the first BAC measure was obtained 15 minutes following beverage consumption. Subjects were also blinded to the beverage type. All subjects were asked to guess which beverage they had received 15 minutes after beverage consumption and all subjects correctly guessed which beverage they had received.

### BAC and CO Measurements

BAC was measured with an Alco-Sensor FST (DAVTECH Analytic Services, Canada) at the beginning of all study visits and at 15 min intervals following beverage consumption (except during PAS administration). A Micro + ™ Smokerlyzer® CO monitor (Bedfont Scientific Ltd.) was used to obtain CO measures at the beginning of all study visits.

### Neurophysiological Assessment

#### TMS Stimulation

TMS pulses were administered to the left motor cortex to obtain the resting motor threshold and 1 mV intensity and to the left DLPFC (for PrePAS, PAS and PostPAS) using a 7 cm figure-of-eight coil, and a Magstim 200 stimulator (Magstim Company Ltd., UK) connected via a Bistim module and electromyography (EMG) data was collected using dedicated software (Cambridge Electronics Design, UK). The RMT was determined according to the protocol outlined by Rossini *et al*., 1994^23^. The RMT was defined as the minimum stimulus intensity that elicits a MEP of more than 50 µV in five of ten trials. The intensity of stimulation was estimated based on the RMT (120% RMT) from the left motor cortex. The intensity of stimulation was then adjusted as necessary to produce a mean peak-to-peak MEP amplitude of 1 mV^24^ in the left motor cortex. In stimulating the left motor cortex, the TMS coil was placed at the optimal position for eliciting MEPs from the right abductor pollicis brevis (APB) muscle. EMG was captured by placing two disposable disc electrodes over the right APB muscle. The signal was amplified using a Model 2024F amplifier (Intronix Technologies Corporation, Bolton, Ontario Canada). The signal was filtered at band pass of 2 Hz to 2.5 kHz and digitized using the Micro 1401 (Cambridge Electronics Design, Cambridge UK).

#### Cortical Evoked Activity

CEA was measured using EEG signals acquired through a 64-channel Synamps 2 EEG system. All electrodes were referenced to an electrode positioned posterior to the Cz electrode. EEG signals were recorded using DC and a lowpass filter of 100 Hz at 20 kHz sampling rate^25^, (see Supplementary Materials and Methods for description of EEG cleaning and analysis). To quantify the PAS-induced potentiation for each session, we calculated the ratio of TMS-evoked potential (TEP) power at each time post-PAS over pre- PAS responses. This ratio was used as an index of potentiation. As the post-PAS timing of maximum potentiation of CEA could vary among subjects, we selected the maximum CEA ratio for each subject after PAS. To assess PAS-induced potentiation in the DLPFC, the 4 left frontal electrodes encompassing the DLPFC (F1, F3, F5 and F7) were used. To assess global PAS-induced potentiation, all 62 electrodes were averaged.

#### Paired Associative Stimulation

PAS was administered to the DLPFC in accordance with the protocol first described by Rajji *et al*., 2013^17^. Briefly, PAS administration consisted of 180 TMS stimuli delivered over the F5 electrode at a frequency of 0.1 Hz. The TMS stimuli over the DLPFC were preceded by peripheral nerve stimulation (PNS) delivered to the right median nerve by 25 ms. Electrical median nerve stimulation was delivered at 300% of the sensory threshold. The sensory threshold was identified as the minimum detectable PNS stimulus. As described by Rajji *et al*., 2013, given that the post timing for maximum potentiation varies among subjects, the maximum ratio for CEA for each subject following PAS was selected^17^. One outlier (with CEA values 3 standard deviations above the mean during the placebo visit) was removed from CEA calculations.

#### Attention

Attention was assessed by having subjects attend to their hand and count the total number of stimulations delivered. Subjects were asked to report the count randomly throughout the 30 minute session and subjects were asked for a final count at the end of the PAS session.

#### Theta-Gamma Coupling

The analysis of coupling of theta-phase and gamma amplitude was performed as described by Rajji *et al*. 2013^17^. Briefly, an entropy based modulation index (MI) was used to quantify coupling: *MI* = [*log*(*N*) − *H*(*P*)]/*log*(*N*)] (see Supplementary Materials and Methods for description of theta-gamma coupling analysis). The choice of the post-PAS time-point for the analysis of theta-gamma coupling for each subject was based on the maximum time of potentiation.

#### Statistics

Statistical analyses were performed using IBM SPSS Statistics (Version 22). Beverage effects on potentiation and theta-gamma coupling were analyzed using a general linear model repeated measures (ANOVA) with beverage (alcohol versus placebo) as the within-subjects factor. For evaluation of mean potentiation, potentiation from all Post-PAS time-points were averaged (Post 0, Post 15, Post 30 and Post60). For evaluation of maximum potentiation, the time point with the maximal potentiation index was selected. Post-hoc analyses were conducted using paired t-tests. Mean 1 mV peak-to-peak intensities (TMS test stimulus intensity) before and after beverage were compared using paired t-tests.

## Results

### 1 mV Intensity (% stimulator output)

A repeated measures ANOVA was used examine if the alcohol or placebo beverage had an effect on 1 mV peak-to-peak intensity between T1 and T2. There was no significant effect of time (F = 1.431; df = 1,14, p = 2.51) and no significant beverage by time interaction effect (F = 3.332; df = 1,14, p = 0.573) (Table 2), suggesting that neither alcohol or placebo beverage has an effect on corticospinal excitability at baseline.

**Table 2:** Experimental characteristics for PAS.

	Alcohol T1	Alcohol T2	Placebo T1	Placebo T2
Resting motor threshold (% stimulator output)	58 ± 8	—	59 ± 8	—
1 mV Intensity (% stimulator output)	71 ± 12	72 ± 14	71 ± 11	71 ± 11
Peripheral nerve sensory threshold (mA)	—	2.6 ± 0.92	—	2.2 ± 0.83
Mean number of sensory stimuli detected (total)/180	—	174 ± 11	—	169 ± 19

^*^Values are in Mean ± 1 Standard Deviation (SD)

### Breath Alcohol Concentration

The mean peak blood alcohol concentration (BAC) was 23.6 mM ± 4.1 mM (range 18.5 mM–34.2 mM). BAC was always at 0 mM at SI_1mVT1_ and peaked during T2. BAC remained above 17.4 mM (the legal intoxication level), with the exception of two subjects who were just below this level during PAS administration.

### Attention

Attention has been shown to affect neuroplasticity^26^. Paired t-tests revealed no significant difference in number of sensory stimuli detected during PAS in the alcohol and placebo conditions, suggesting that attention levels were not significantly different between the two conditions (t = −0.946;df = 14; p = 0.360) (Table 2).

### Effect of Beverages on Cortical Evoked Activity

**Figure 2:**
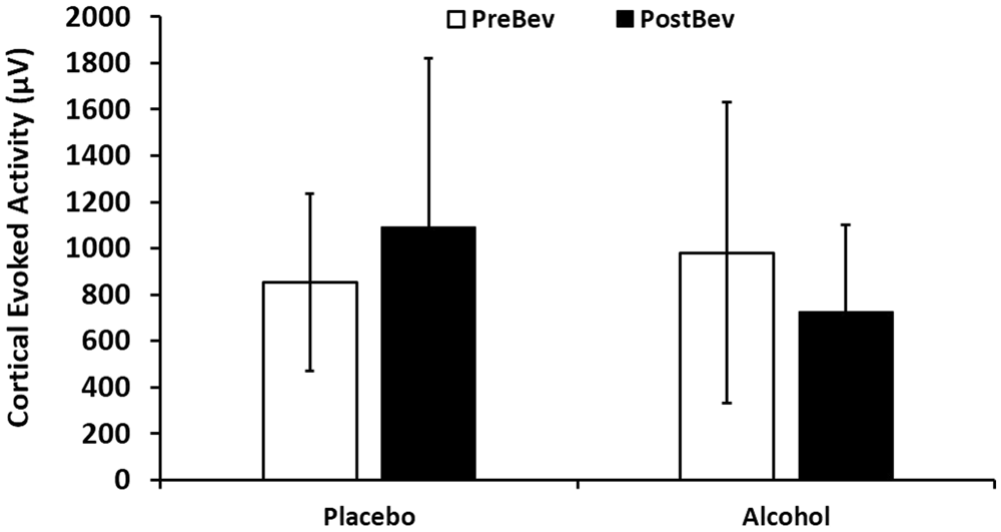
Cortical evoked activity (CEA) before (white bars) and after (black bars) beverage (μV) for the placebo and alcohol conditions (n = 14). Error bars represent the standard deviations. Neither placebo or alcohol beverage produced a significant change in CEA in the DLPFC compared to before beverage. Following beverage consumption, there was a significant difference between cortical evoked activity in the alcohol and placebo groups.

A repeated measures ANOVA revealed a significant beverage by time interaction effect on cortical evoked activity (F = 5.919; df = 1,13, p = 0.030). No significant effect of beverage (F = 3.451; df = 1,13, p = 0.086) or time (F = 0.11, df = 1,3, p = 0.920) was observed. Paired t-tests revealed that neither the placebo beverage (t = −1.641, df = 13, p = 0.125) or alcohol beverage (t = 2.054, df = 13, p = 0.061) produced a significant change in CEA in the DLPFC compared to before beverage. Paired t-tests also revealed that there was also no significant difference in cortical activity before beverage between the placebo and alcohol conditions (t = −1.263, df = 13, p = 0.229). Following beverage consumption, there was a significant difference between cortical evoked activity in the alcohol and placebo groups (t = −2.696, df = 13, p = 0.018). These findings suggest that acute alcohol consumption produces a decrease in cortical evoked activity (Fig. 2).

### Effect of Alcohol on Neuroplasticity in the DLPFC

**Figure 3:**
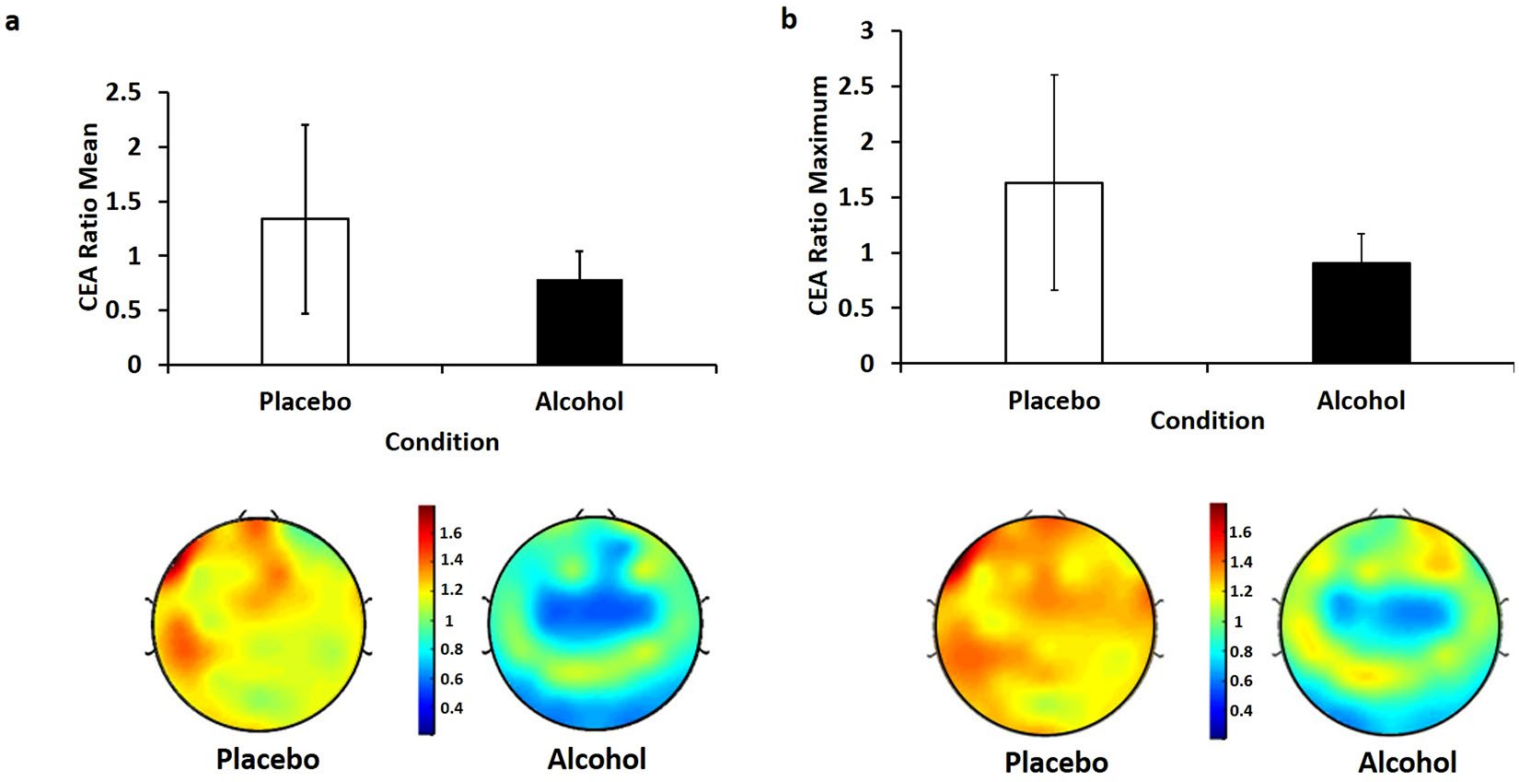
(**a**) Mean ratio of 100 TMS pulses to the DLPFC across all post-PAS timepoints (Post 0 min, Post 15 min, Post 30 min, Post 60 min) to 100 TMS pulses to the DLPFC pre-PAS for the placebo (white bar) and alcohol(black bar) conditions (n = 14). Error bars represent the standard deviations. Alcohol significantly impaired mean PAS-induced neuroplasticity. (**b**) Mean ratio of 100 TMS pulses to the DLPFC at time point of maximum potentiation compared to 100 TMS pulses to the DLPFC pre-PAS for the alcohol(black bar) and placebo (white bar) conditions (n = 14). Error bars represent the standard deviations. Alcohol significantly impaired maximal PAS-induced neuroplasticity. The panels on the bottom represents average topoplots of alcoholcompared to placebo, with hotter colours representing greater CEA following PAS.

The repeated measures ANOVA revealed a main effect of beverage (F = 6.034; df = 1,13, p = 0.029), reflective of decreased PAS-induced neuroplasticity in the DLPFC, compared to placebo. Alcohol intoxication resulted in a significant impairment of the mean potentiation compared to placebo beverage (t = 2.456, df = 13, p = 0.029) in the DLPFC (Fig. 3). Alcohol intoxication also resulted in impaired peak PAS-induced neuroplasticity in the DLPFC compared to the placebo beverage, as indexed using the mean maximum CEA ratio in the DLPFC (t = −2.945, df = 13, p = 0.011; Fig. 3). Alcohol significantly impaired mean (t = −3.051, df = 13, p = 0.009) and maximum DLPFC PAS-induced neuroplasticity globally (t = −3.260, df = 13, p = 0.006). A one sample t-test confirmed that potentiation occurred under the placebo condition, as the mean maximum CEA ratio was significantly greater than 1 in the DLPFC (t = 2.432, df = 13, p = 0.030) and globally (t = 2.325, df = 13, p = 0.037).

### Alcohol’s Effects on Theta-Gamma Coupling

**Figure 4:**
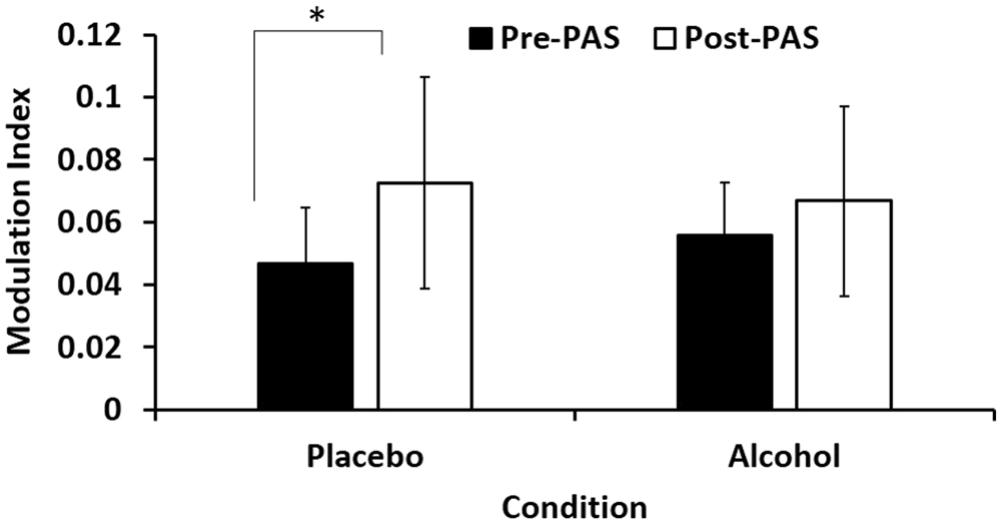
Theta-gamma coupling. Theta-gamma coupling is indexed through the modulation index (MI). There was a significant increase in MI following PAS with placebo beverage. This significant increase in MI was not observed following PAS with the alcohol beverage. Error bars represent the standard deviations.

An exploratory analysis was conducted to explore whether PAS had an effect on theta-gamma coupling. The repeated measures ANOVA revealed a significant effect of time (PrePAS vs max potentiation time Post-PAS), reflective of increased theta- gamma coupling following PAS (F = 7.516, df = 1,14, p = 0.016). No significant effect of beverage (F = 0.111, df = 1,14, p = 0.744) or beverage by time interaction was (F = 2.720, df = 1,14, p = 0.121). Paired t-tests revealed that PAS to the DLPFC resulted in an increase in MI, indicating increased theta-gamma coupling during the placebo visit (t = 2.954, df = 14, p = 0.010). This significant increase following PAS was not observed during the alcohol visit (t = 1.486, df = 14, p = 0.159) (Fig. 4).

## Discussion

Impairment of mean and maximum potentiation from this study indicate that alcohol intoxication significantly impairs PAS-induced neuroplasticity in left DLPFC and globally. The impairment of neuroplasticity was accompanied by impairment of the PAS-induced increase in theta-gamma coupling in the left DLPFC. These findings provide a possible mechanism by which alcohol intoxication impairs cognitive function.

### Alcohol’s Impairment of Neuroplasticity in the DLPFC

Intoxication by alcohol impaired PAS-induced neuroplasticity in the left DLPFC and globally. Previously, it has been demonstrated that a single heavy drinking episode^11^ and even low doses of alcohol^10^ impair PAS-induced neuroplasticity in the motor cortex. Using DLPFC PAS and EEG, current findings demonstrate that this impairment of neuroplasticity by alcohol intoxication occurs in DLPFC. Interestingly, we found that the impairment in neuroplasticity induced by alcohol was not just localized to the DLPFC. Rather, we also observed a global impairment of neuroplasticity. It is not well understood whether this global impairment of neuroplasticity is a result of neuroplasticity induction within regions outside the DLPFC or a phenomena secondary to modulation of neuroplasticity in the DLPFC. Given that the DLPFC is functionally connected to a number of cortical and subcortical regions, the global effect observed may be due to downstream neuroplasticity mechanisms. Global evoked response potential (ERP) deficits have been reported in binge drinkers compared to non-drinkers during basic and high level cognitive states^27^. Global aberrancies in neuronal responses may highlight neural inefficiencies in heavy drinkers (as subjects in the study reported at least one previous binge drinking episode in the last month) that contribute to a less localized induction of neuroplasticity in this population. Induction of less localized neuroplasticity in this population along with alcohol’s widespread action on the brain^1^ may explain the global impairment of neuroplasticity seen in the present study. Interestingly, we observed that alcohol produced a suppression of CEA following beverage consumption, suggesting that alcohol suppresses both CEA and PAS-induced neuroplasticity.

### Mechanisms for Alcohol’s Impairment of Neuroplasticity

The mechanisms of alcohol’s impairment of neuroplasticity in the DLPFC is likely related to its antagonistic effect on NMDA receptor mediated neurotransmission and potentiating effect on GABA_A_ receptor mediated neurotransmission^28–31^. Using paired pulse TMS paradigms, Ziemann *et al*., 1995 demonstrated that alcohol consumption results in an increase of TMS indices of GABA_A_ and GABA_B_ receptor mediated inhibition and a decrease in TMS indices of NMDA receptor mediated excitability in healthy subjects^30^. Studies using rat hippocampal slices have demonstrated that both GABA_A_ and NMDA receptors are necessary for the complete reduction of neuroplasticity by alcohol. Pharmacological isolation of NMDA receptors by antagonism of GABA_A_ receptors via picrotoxin did not produce the complete impairment of neuroplasticity otherwise produced by alcohol in hippocampal slices. Rather, alcohol produced greater impairments of LTP when GABA_A_ receptors were functional (as seen in the absence of picrotoxin)^29^. Taken together, these studies reveal that both GABA_A_ and NMDA receptor activity contribute to the neuroplasticity deficits produced by alcohol.

### Alcohol’s Impairment of Theta-Gamma Coupling

An increase in theta-gamma coupling following PAS with placebo beverage was observed in the present study. These findings are consistent with Rajji *et al*., 2013 who demonstrated that PAS potentiated theta-gamma coupling in the DLPFC among healthy subjects. Interestingly, this increase in theta-gamma coupling following PAS was not observed with alcohol. Theta-gamma coupling is believed to be an index of DLPFC functioning, specifically working memory^18, 32^. Theta-gamma coupling has been shown to be increased during trials that required ordering information compared to trials that do not require ordering^17^. Previous studies have demonstrated that acute alcohol intoxication produces an impairment of working memory across several domains^33–35^. Increased theta-gamma coupling following PAS observed in the current study is speculated to be due to PAS activating the same neuronal networks in the DLPFC involved in working memory^17^. Therefore, alcohol’s impairment of PAS induced potentiation of theta-gamma coupling may be associated with alcohol’s effects on neuroplasticity in neuronal networks involved in learning and memory.

### Implications for Cognitive and Executive Function

Current findings demonstrate that alcohol intoxication impairs PAS-induced neuroplasticity in the DLPFC, a brain region that plays an important role in cognitive and executive functioning. Finding of alcohol’s impairment of potentiation of theta-gamma coupling suggest that alcohol intoxication impairs neuroplasticity in neuronal networks involved in cognitive functioning. Impaired cognitive function is a commonly reported effect of alcohol intoxication^36^. The BAC level required to impair cognitive function varies with the type of task. Complex cognitive tasks (ie. tasks requiring divided attention) are impaired at BACs as low as 0.01%^37^. At higher BACs, as seen following a heavy drinking episode (≥0.08%BAC), a much wider range of cognitive impairments are seen, including a slowing of simple reaction time^38, 39^. The impairment of neuroplasticity in the DLPFC observed in the present study provides a potential mechanism by which alcohol intoxication impairs cognitive function.

Given that the DLPFC is a part of the brain reward circuitry as a part of the mesocortico-limbic pathway, impairment of neuroplasticity in the reward circuitry by alcohol may also play a role in the transition to alcohol use disorders (AUDs) following repetitive heavy drinking. The DLPFC regulates the integration of goal-motivated behavior by assimilating information regarding the potential negative and positive outcomes of selecting a behavior. Aberrant neuroplasticity in this regions result in the selection of inappropriate behaviors despite their negative consequences, such as compulsive drug taking^40^. Indirect evidence for impaired neuroplasticity in the DLPFC of alcohol dependent individuals comes from findings of neuromodulatory brain stimulation studies that have demonstrated that modulation of neuroplasticity in the DLPFC through repetitive transcranial magnetic stimulation shows promise as a treatment of AUDs^41, 42^. Identifying the existence, localization and direction of neuroplasticity impairment in the DLPFC of alcohol dependent individuals through PAS with EEG can help inform the future use of neuromodulatory brain stimulation for the treatment of individuals with AUDS.

The present study has a number of limitations. Firstly, it would have been informative to examine the effect of alcohol intoxication on working memory. This would have allowed us to determine if alcohol’s impairment of neuroplasticity and theta-gamma coupling is associated with working memory dysfunction. However, given that theta-gamma coupling has been demonstrated to be related to working memory load, we can infer that alcohol is acting on the same neuronal networks involved in working memory to impair potentiation of theta-gamma coupling. Another limitation to the current study is that we used breath samples to calculate BAC levels rather than measuring from blood samples. However, it would have been unnecessarily invasive and disruptive to take blood samples from patients throughout the beverage visits as breath measures correlate highly with BAC^43^. Another limitation of the study was the sample size of the present study is relatively small. However, the within-sample design of the study allowed for the use of the current sample size. Lastly, while we attempted to blind the subjects to their beverage type, all subjects correctly guessed which beverage they had received during both beverage visits. However, we have no reason to believe that being aware of which beverage they had received would affect their neurophysiological measurements and neuroplasticity.

## Conclusions

Findings from the current study revealed that alcohol intoxication impaired neuroplasticity in the DLPFC and throughout the cortex. Additionally, the increase in coupling of the theta-gamma coupling following PAS was not observed following alcohol consumption. As theta-gamma coupling is believed to be related to working memory, these findings provide a potential mechanism for impairment of cognitive function by alcohol. Furthermore, as the DLPFC is a part of the brain reward circuitry, impairment of neuroplasticity in this region may play a role in the transition to AUDs following repetitive heavy drinking. Future research is needed to determine the role of DLPFC neuroplasticity in the pathophysiology of AUDs.

## Electronic supplementary material

Supplementary Material and Methods
