# Supplementary material for: Impairment of Neuroplasticity in the Dorsolateral Prefrontal Cortex by Alcohol
Source: Sci Rep. 2017 Jul 13;7:5276. doi: 10.1038/s41598-017-04764-9 (PMC5509647; doi:10.1038/s41598-017-04764-9)
Supplement: Supplementary file 1 — Supplementary Material and Methods [file 41598_2017_4764_MOESM1_ESM.pdf]

Impairment of Neuroplasticity in the Dorsolateral Prefrontal Cortex By Alcohol

Genane Loheswaran, MSc
Mera S. Barr, PhD
Reza Zomorrodi, PhD
Tarek K. Rajji2,4, MD, FRCP(C)
Daniel M. Blumberger, MD, MSc, FRCP(C)
Bernard Le Foll, MD, PhD, MCFP (C)
Zafiris J. Daskalakis, MD, PhD, FRCP(C)

Supplementary Materials and Method

CEA from DLPFC

EEG data were down-sampled to 1000Hz and segmented from -1000ms to 2000ms relative to the onset of the TMS pulse. The data was then baseline corrected with respect to the pre-stimulus interval -500 ms to -110 ms. EEG data were then re-segmented from 25ms to 2000ms. Thereafter, the EEG data was digitally filtered by using a second order, Butterworth, zero-phase shift 1-55 Hz band pass filter (24dB/Oct). In order to apply the same objective and subjective criteria to de-noise data, the EEG recordings from all 5 sessions (PrePAS, Post0, Post 15, Post 30 and Post 60) were concatenated together. Initially, EEG data were visually inspected to eliminate trials and channels that were highly contaminated with noise. Then, an electrodes-by-trials matrix of ones was created and assigned a value of zero if an epoch had: (1) an amplitude larger than +/- 150 μV; (2) a power spectrum that violated the 1/f power law; or (3) a standard deviation 3 times larger than the average of all trials. Additionally, electrodes were rejected if their corresponding row had more than 60% of columns (trials) coded as zeros and epochs were removed if their corresponding column had more than 20% of rows (electrodes) coded as zeros. Lastly, an independent component analysis (ICA) (EEGLAB toolbox; Infomax algorithm) was performed to remove noise from the EEG data and data was re-referenced to the average for further analysis.

To assess potentiation of CEA by PAS, first, the TMS evoked potential (TEP) for each session was calculated by averaging the response over all epochs. Next, using the Hilbert transformation, the area between 50-275 ms under the instantaneous amplitude of TEP was determined. The first interval (i.e., 50 ms) was chosen because it represents the earliest artifact-

free data and the second interval (i.e., 275 ms) was chosen to cover the activity of $GABA_B$ receptors [1,2].

Theta Gamma Coupling

The analysis of coupling of theta-phase and gamma amplitude was performed on the time averaged response of the TMS evoked potential of each participant using Matlab. The averaged signal was first filtered into separate theta [4-7]Hz and gamma [30-50]Hz waveforms with a zero-phase shift filter and thena Hilbert transform was applied to separate the phase and amplitude of the signal. The gamma amplitudes were sorted into six bins (i.e., _180° to _120°, _120° to _60°, _60° to 0°, 0°–60°, 60°–120°, 120°–180°) using the phase information of the theta wave and were then averaged. Given that the angle values correspond to the cosine reference, the peak of the waveform falls at zero degrees. An entropy based modulation index (MI) was used to quantify coupling: $MI=[log(N)-H(P)]/log(N)]$

Where N is the number of phase bins, log(N) represents the entropy of a uniform distribution, P is the relative amplitude distribution sorted according to phase bins, and H(P) is the entropy of the P distribution, which is calculated as:

$H(P)= -\sum^{N}_{j=1} P(j) \log [P(j)]$

The relative amplitude distribution P for each participant was calculated by dividing the amplitude of each phase by the sum of all amplitudes across bins. The maximum entropy for a relative amplitude distribution happens when the amplitude is 1/N, which occurs when the distribution is uniform. Given that an increase in coupling represents an increase of order, higher coupling translates to lower entropy H(P). This in turn produces a high MI value. The choice of the post-PAS time-point for each participant was based on the maximum time of potentiation.

1 Deisz, R. A. GABA(B) receptor-mediated effects in human and rat neocortical neurones in vitro. *Neuropharmacology* **38**, 1755-1766 (1999).
2 Deisz, R. A. The GABA(B) receptor antagonist CGP 55845A reduces presynaptic GABA(B) actions in neocortical neurons of the rat in vitro. *Neuroscience* **93**, 1241-1249 (1999).
